# *Listeria monocytogenes* in organic and conventional farming: Epidemiology, risks, and solutions within a One Health framework^☆^

**DOI:** 10.1016/j.onehlt.2025.101173

**Published:** 2025-08-19

**Authors:** E. Ryzhova, Wichmann Janine, Howlett-Downing Chantelle, O. Holý

**Affiliations:** a Science and research Centre, Faculty of Health Sciences, Palacký University Olomouc, Czechia; b School of Health Systems and Public Health, University of Pretoria, South Africa; c South African Medical Research Council, Environmental Health Unit, Pretoria, South Africa

**Keywords:** *Listeria monocytogenes*, One Health, Farming, Surveillance

## Abstract

*Listeria monocytogenes* is a resilient, zoonotic pathogen that poses significant challenges across human, animal, plant, and environmental health systems. This review explores the epidemiology of *L. monocytogenes* within the One Health framework, emphasizing its transmission dynamics, risk factors, and implications for food safety. The pathogen's ability to persist in diverse environments, form biofilms, and withstand extreme conditions highlights its role as a major public health concern, particularly for vulnerable populations such as pregnant women, immunocompromised individuals, and the elderly.

The review examines the intersection of organic and conventional farming practices with *L. monocytogenes* contamination, noting the unique risks associated with organic fertilizers, wildlife exposure, and limited antimicrobial interventions. In contrast, conventional systems face challenges such as crowded animal housing and antimicrobial resistance. The role of plants as vectors, particularly through contaminated soil, irrigation water, and fertilizers, is underscored, with a focus on the risks linked to minimally processed and ready-to-eat foods.

Environmental reservoirs, including soil, water, and biofilms, are identified as critical contributors to the pathogen's persistence and transmission. Climate change, agricultural practices, and industrial processes further exacerbate the complexity of *L. monocytogenes* control, necessitating cross-disciplinary approaches.

The review concludes with a call to strengthen the One Health framework through integrated surveillance, sustainable farming practices, public awareness campaigns, and innovative technologies. By addressing the multifaceted challenges posed by *L. monocytogenes*, this approach aims to ensure food safety, promote ecological sustainability, and protect public health in an increasingly interconnected and climate-impacted world.

## 1 Introduction

The “One Health” concept views humans, animals, plants, and the environment as inseparable systems. Human and animal health depend on each other and are linked to the health of ecosystems. Food safety is inextricably linked to micro and macroeconomics, sustainable development, personal agency, purchasing habits, commercial and traditional farming methods [1]. In order to ensure a sustainable dynamic between these interdependent systems, the “One Health” concept was developed. The concept is particularly applicable for preventing the spread of zoonotic diseases [2]. Interactions between humans, animals, and plants may lead to the emergence of new or modified pathogens, accelerate their spread, and increase the risk to susceptible organisms.

Ecological initiatives for improving the environment often align with the “One Health” concept. One such initiative is ecologically clean, organic farming.

Organic farming is defined as an agricultural system that emphasizes the use of natural processes and materials (waste). By incorporating these techniques into the farming ecology, the methods avoid using synthetic chemicals like pesticides and fertilizers. Further organic techniques include crop rotation, composting, companion planting and biological pest control. This holistic approach aims to enhance soil fertility, promote biodiversity, and maintain ecological balance. However, many of the animal sources of such amendments (e.g., cattle, sheep, and poultry) are known sources of foodborne pathogens that are often linked to fresh produce outbreaks, such as *Escherichia* (*E.*) *coli* O157:H7, non-O157 Shiga toxin-producing *E. coli* (STEC), *Salmonella* spp., and *Listeria* (*L*.) *monocytogenes* [3].

Although consumer interest in organic products is rising [4,5], evidence linking organic food consumption to listeriosis risk remains inconsistent. Cohort studies by Mylonakis et al. (2002) [6], Charlier et al. (2017) [7], and Coipan et al. (2023) [8] have provided critical insights into risk factors for listeriosis in vulnerable populations, including pregnant women, the elderly, and immunocompromised patients, but none demonstrated a clear link between organic food consumption and increased listeriosis incidence. These studies underline the complexity of attributing individual listeriosis cases to specific food sources and highlight the need for integrated surveillance. The WHO/FAO report on *L. monocytogenes* in ready-to-eat foods also emphasizes the importance of source attribution, strain characterization, and systematic monitoring across food systems [9].

Adherents of such ecological initiatives often overlook that humans have long altered farming processes to meet the demand for large-scale production. Chemical antiseptics, reagents, antibiotics, and fertilizers have been employed to ensure sufficient quantities of healthy produce for growing human populations. Given these anthropogenic changes in ecosystems, the safety of returning to organic farming today remains uncertain. The safety of ecologically clean products has economic and health benefits.

**Fig. 1:**
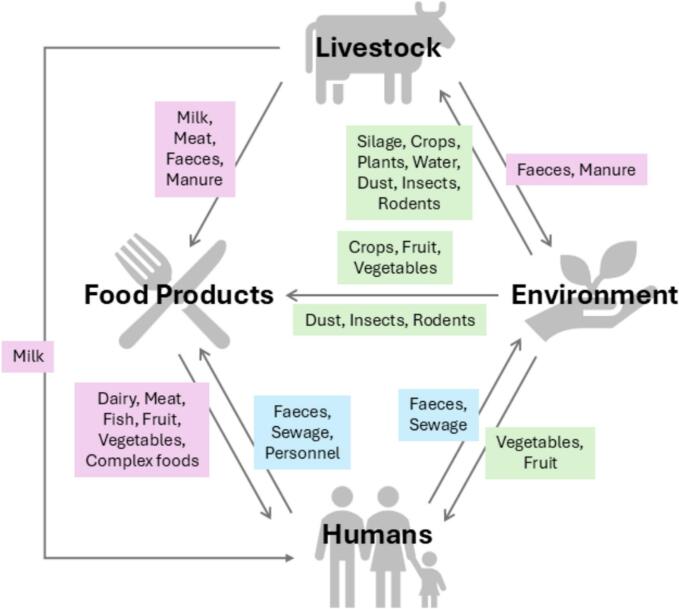
Transmission routes for *L. monocytogenes* (modified from Walland et al. 2015) [10].

Zoonotic infectious agents pose a particularly complex challenge (Fig. 1.; Table 1). These pathogens successfully parasitize humans and animals while thriving in diverse environmental conditions. Understanding the epidemiology of zoonotic infections requires a thorough investigation of pathogen habitats and transmission routes. However, there is no consensus among researchers regarding the safety of environmentally friendly, organic farms with respect to the spread of zoonotic infectious agents. This review addresses this issue through the example of *Listeria monocytogenes*, the pathogen that causes listeriosis.

**Table 1:** Summarizes selected studies reporting the prevalence of *L. monocytogenes* on organic and conventional farms. The data illustrate high variability across regions and farming systems*.*

Study	Location	System	Prevalence of *L. monocytogenes* (%)	Notes
Esteban et al. (2008) [11]	Spain	Free-range	13.5	Samples from free-range poultry farms
Jones et al. (2012) [12]	USA	Organic & conventional	26.5 (organic) / 26.7 (conventional)	Prevalence on laying hen farms
Wilhelm et al. (2009) [13]	Canada	Organic dairy	14.0	Organic dairy farms; no direct comparison with conventional
Varsaki et al. (2022) [14]	Spain	Conventional dairy	11.5	Conventional dairy farms with dairy cattle

The objectives of this literature review are twofold: (1) to analyse published research on listeriosis within the One Health framework and (2) to propose a methodology for assessing the risks of *L. monocytogenes* contamination in both organic and conventional farm products.

The review is structured according to the One Health framework, focusing on findings related to trends in *Listeria* spp. infection dynamics and their connection to organic farming practices.

## 2 Listeria monocytogenes

*L. monocytogenes* is a gram-positive, facultative intracellular bacillus that is transmitted primarily via the alimentary route [15]. Listeriosis is among the leading causes of mortality from foodborne infections [16], with symptoms ranging from sore throat to meningoencephalitis and septicaemia [17]. This pathogen poses significant risks, particularly to vulnerable groups, with case fatality rates exceeding 20 % [18]. *L. monocytogenes* is widely distributed in the environment and can infect both wild and domestic animals [19].

*L. monocytogenes* is characterized by its resilience under adverse conditions. It can survive high salt concentrations, low pH levels (4.7–9.2), and extreme temperatures below 0 °C and above 40 °C [20,21]. Its ability to grow at subzero temperatures, starting at −0.3 °C, was demonstrated by Junttila et al. [21], who also reported that pathogenic strains thrive better in cold conditions than nonhemolytic strains. Growth is optimal up to 35 °C, but viability decreases significantly at relatively high temperatures [22]. Another notable feature of *L. monocytogenes* is its long incubation period, which can last up to 70 days. This lengthy latency complicates source identification, hindering efforts to prevent new infections [23].

## 3 People

Healthy adults, excluding pregnant women, rarely develop listeriosis [24]. However, 90 % of cases occur in at-risk populations [25], and this group has significantly expanded. Several risk factors for listeriosis infection have been identified.

Age is a leading risk factor for listeriosis infection. For example, between 1990 and 2005 in the US, individuals over 65 years of age accounted for almost 70 % of listeriosis deaths, with the highest mortality rate among those over 85 years of age [26]. Various immunodeficiency conditions, including cancer, increase susceptibility to listeriosis. Patients with chronic lympholeukemia are 1000 times more likely to be infected than healthy individuals [27]. At the University of Texas M. D. Anderson Cancer Center, the incidence of listeriosis was 191 cases per 100,000 among patients with a primary haematological malignancy and 21 cases per 100,000 patients with a primary solid tumour [28]. Advanced-stage cancer and chemotherapy exacerbate risk, as illustrated by a hospital outbreak linked to contaminated sandwiches from the hospital cafeteria [29]. Listeriosis frequently occurs alongside acquired immunodeficiency syndrome [26,30,31].

The rise in iatrogenic immunosuppression therapies, such as biologics, cytostatics, and corticosteroids, has increased the risk of listeriosis [32,33]. Bodro and Paterson [34] reported 228 cases of listeriosis linked to biologic therapy over eight years. Similarly, Skogberg et al. [35] reported that 43 % of 74 listeriosis cases in Helsinki involved immunosuppressive therapy, such as monoclonal antibodies or high-dose methylprednisolone. Listeriosis is most often observed in patients who are on a combination of two or more immunosuppressive drugs and who are receiving oncological treatment or renal transplantation [36]. Hospital outbreaks tied to ready-to-eat sausages have shown high infection rates (10/11) among patients receiving combined immunosuppressant steroid hormones and proton pump inhibitors [37].

Listeriosis incidence increases during seasonal viral infections, such as influenza and gastroenteritis, which disrupt the mucoid layer of the gastrointestinal tract [37,38]. Listeriosis infections also seem to be more common during hospitalizations. In Rio de Janeiro, for example, the incidence of listeriosis was 0.53 cases per 1000 hospital admissions, with an average of nine days in the hospital [39]. Silk et al. [40] identified 30 reports of foodborne listeriosis in hospitals in 13 countries, mostly in major cities in the US or Europe.

Among healthy adults, pregnant women seem to be most susceptible to *L. monocytogenes.* In Central Asia, every 10th pregnant woman is infected with *L. monocytogenes* [41]. In the U.S., pregnant women have a 17-fold greater risk of listeriosis than nonpregnant women [6]. Although pregnant women experience mild symptoms, complications include miscarriage or stillbirth. Moreover, *L. monocytogenes* may persist in the human faecal microbiota and vaginal microflora, contributing to environmental contamination [42,43]. Recent evidence also indicates that faecal carriage of *L. monocytogenes* is relatively common and strongly influenced by the composition of the gut microbiota [44]. For pregnant women, risk factors include the consumption of raw milk, cheese, and farm products, which are prone to *L. monocytogenes* contamination [45].

At-risk populations for *L. monocytogenes* are growing. The susceptibility of ageing populations in many countries is increasing [46]. Similarly, the widespread use of immunosuppressive therapies creates new vulnerabilities and hospital-acquired listeriosis remains a challenge, with outbreaks linked to contaminated ready-to-eat foods. People have also become more interested in organic and healthy foods, perceiving these foods as healthier [4,5]. Several studies have also reported chronic vaginal and intestinal carriage [42,46]. Despite a growing interest in organic and ready-to-eat food, such as sandwiches and prepacked salads [47,48], we could not find any studies revealing a correlation between these foods and listeriosis infection.

## 4 Animals

Listeriosis affects wildlife, domestic animals, and birds [49], with *L. monocytogenes* recognized as a zoonotic agent maintaining a continuous life cycle between animals and the environment [14]. Synanthropic animals, which live at the interface of human-altered environments and wildlife, play a significant role in the transmission of zoonotic infections. They act as reservoirs, vectors, and susceptible hosts. For example, *L. monocytogenes* has been isolated from foxes and martens in Poland [50], and flies collected near restaurant waste in Washington, D.C. [51].

Listeriosis is generally nonfatal in wild or domestic animals. Many infected animals remain asymptomatic while excreting the pathogen [52,53]. The bacterium has been detected in the feces, nasal, and genital secretions of healthy animals [54]. Asymptomatic infections prevent the isolation and treatment of farm animals while facilitating infection spread in the herd, habitat, and livestock pens. Chronic asymptomatic intramammary infections in goats and subclinical mastitis in sheep and cows have also been linked to *L. monocytogenes*, with contaminated milk and cheese posing risks to humans [55,56]. Infected milk may appear normal, and animals often show no systemic symptoms, complicating detection and containment.

Farming practices may influence the spread of *L. monocytogenes*. However, no large-scale studies have compared microbial loads on farms with different farming styles. Most of the available evidence focuses on free-range farms. Free-range systems, while promoting animal welfare, may increase pathogen transmission, as evidenced by the increased mortality of young birds on free-range farms [57]. Studies have shown high faecal contamination of *L. monocytogenes* on free-range farms, potentially transmitted from animals to crops and vice versa [[11], [58]]. According to European guidelines, keeping animals on organic farms requires that at least 60 % of their feed come from the same farm, perpetuating the infection cycle [59].

The rates of listeriosis infection on free-range and conventional farms seem to be comparable, with crowding and poor sanitation in traditional systems correlating with increased listeriosis prevalence [54]. Moreover, on traditional farms, where animals are permanently housed in enclosures and cages, infection rates range from 11 % in the UK [60] to 15 % in Kazakhstan [61]. Antibiotic use on conventional farms results in antimicrobial-resistant strains, but resistance levels between organic and conventional systems show no consistent differences [[14], [62],^63^].

The relative risk of listeriosis between organic, free-range and conventional farms remains unclear. Some studies report no significant differences in prevalence, whereas others highlight heightened risks on organic farms owing to limited antimicrobial use and greater exposure to wild animals [[13], [64]]. Jones et al. [12] reported that the prevalence of *Listeria* spp. on U.S. farms was extremely low, with similar prevalence rates on organic and conventional farms. In contrast, the prevalence of *Listeria* spp. was similar on organic and conventional farms, at 26.5 % and 26.7 %, respectively [11].

Determining infection rates across farming systems necessitates comprehensive, region-specific studies comparing organic and conventional farms of varying types. Only through such analyses can we accurately assess whether free-range or traditional farming systems pose greater risks for *Listeria* spp. transmission.

## 5 Plants

*L. monocytogenes* exhibits significant biological adaptability, infecting both animal products and plants. Truong et al. [65] noted that any plant can serve as a habitat for *L. monocytogenes*. Contamination typically arises from contact with contaminated soil, fertilizers, or irrigation water [66]. *Listeria* spp. biofilms can colonize fruits and vegetables throughout the food chain, from cultivation to processing [67]. *L. monocytogenes* has even been detected in plants grown in previously pathogen-free fields, suggesting the possibility of seed-mediated transmission [68].

Several human listeriosis outbreaks have occurred because of the consumption of plant products, particularly low-acid fruits. In a 2011 outbreak linked to melon consumption, 147 cases were reported across 28 U.S. states, with a 22 % mortality rate [69]. Ready-to-eat produce stored under refrigeration, such as fruits, herbs, berries and a range of vegetables, poses significant risks. Leafy greens such as lettuce and spinach are less frequently infected than fruits such as tomatoes and cucumbers [3]. Low-acid fruits seem to be more strongly associated with listeriosis. Fruits with a relatively high pH, such as melons, have a greater potential for *L. monocytogenes* film infection than domore acidic fruits such as pears [70]. Similarly, papaya, a low-acid fruit, is a good substrate for *L. monocytogenes* growth [71].

Contaminated feed is the primary source of *Listeria* spp. transmission among animals [72]. Plant-based feed, often unprocessed, provides a suitable environment for *Listeria* spp. Vlasov and Pavlova [73] demonstrated that *Listeria* spp. can colonize and multiply in growing plant tissues. Silage, particularly in bale storage systems with elevated pH values, is another suitable substrate for *L. monocytogenes* growth [74,75].

The microbiological risks associated with organic fertilizers, such as manure, are well-documented [65]. The use of manure as an organic fertilizer can result in vegetables being contaminated with *L. monocytogenes* [76]. *Listeria* spp. can persist in the soil for 128 days after manure application, increasing the potential contamination risk [3]. *L. monocytogenes* has been detected in cabbage [76] and spinach [77]. The contamination of soil affects farm produce and the spread of *L. monocytogenes* to the shoes, clothing, and hands of field workers, potentially infecting workers and their families [78].

The growing demand for minimally processed foods heightens the need for vigilance regarding *Listeria* spp. in plant-based products. Epidemiologists should consider plant contamination alongside traditional risks from milk and unpasteurized dairy products. The relative contamination rates of organic versus conventional produce remain unclear, necessitating further research to guide safety practices for both human and animal food systems.

## 6 Environment

*Listeria* spp. bacteria are widespread in the environment [19]. *L. monocytogenes* has been detected in soil, sea, river and tap water, plants, seeds, silage pits and even algae [49,70]. Contaminated soil and water can serve as reservoirs, spreading *Listeria* spp. to animals, plants, and ultimately humans. Stea et al. [79] reported *Listeria* spp. and *L. monocytogenes* in 53.8 % of surveyed water sources, with higher detection rates (72.1 %) in rural areas than in urban areas (35.4 %).

The soil microbiome, a complex system of bacteria, viruses, and fungi, plays a critical role in inhibiting the spread of *L. monocytogenes* [65]. For example, Moynihan et al. [80] reported that *Listeria* spp. populations decline faster in soils with diverse microbial communities. Compared with conventional farms, organic farms typically host richer, more heterogeneous soil microbiomes [81]. Practices such as crop rotation and intercropping improve soil quality, whereas monoculture weakens microbial diversity [82].

However, external factors often drive soil contamination. For example, Dowe et al. (1997) reported a relatively high *L. monocytogenes* prevalence in plants from unfertilized, uncultivated fields likely due to wild activity and a relatively weak soil microbiome [83]. Climatic conditions also affect the prevalence of *Listeria* spp. As a psychrophilic organism, *Listeria* spp. thrives at low temperatures, with growth observed at temperatures as low as −0.4 °C [12]. Chandler-Khayd et al. [3] linked winter manure application to relatively high *L. monocytogenes* levels in the soil. Stea et al. [79] reported *Listeria* spp. and *L. monocytogenes* in water year-round but observed higher detection rates during colder months due to reduced microbial competition. However, Semenza et al. [84] reported no evidence linking climatic events such as drought or heatwaves to increased *Listeria* spp. outbreaks.

*Listeria* spp. bacteria can survive for years in food processing facilities. *L. monocytogenes* forms biofilms on surfaces such as glass, rubber, and metal surfaces, including refrigeration equipment, making them resistant to cleaning and disinfection [85,86]. These biofilms enhance antibiotic resistance and complicate microbial removal [87]. Schäfer et al. [88] reported high contamination rates in poultry processing equipment, even after cleaning. *Listeria* spp.'s ability to enter a “viable but nonculturable form” further complicates detection [86]. Moreover, clinical strains exhibit greater biofilm-forming ability than those from food facilities, raising concerns about potential hospital colonization [89].

The persistence of *L. monocytogenes* is linked to biofilm resilience and colonization of hard-to-clean surfaces [90]. Effective prevention requires cleaning and disinfection as well as maintaining aseptic conditions to inhibit biofilm formation.

The widespread presence of *Listeria* spp. soil, water, plants, animals, industrial settings, and farms underscores the challenges of controlling its spread. Changing agricultural and production practices, including organic farming and biodiversity promotion, may inadvertently contribute to environmental contamination [91]. The impacts of farm and production conditions on the development of *Listeria* spp. biofilms remain unclear. However, an outbreak linked to organic products could erode consumer trust and jeopardize the European organic program.

## 7 Weather and climate

*L. monocytogenes* has been reported to be ubiquitous within the natural environment; however, several features of the epidemiology and characteristics of the microbe make it especially climate sensitive [92]. Acute increases in ambient temperature and high summer temperature peaks, for example, have been linked to the occurrence of listeriosis and diarrheal pathogens. Another important aspect to consider for food-borne *Listeria* spp. is its presence throughout the food chain — during pre-harvest, processing, and at the retail level. The hygiene and handling of the product by labour in a water constrained region will influence the potential and intensity of a *L. monocytogenes* outbreak.

South Africa is susceptible to impacts of climate change on an already strained national bulk water delivery system [93]. The management of food borne *Listeria* spp. requires public health practitioners to consider the context of current water use practices within the agricultural, industrial and domestic situations where surface water is often used when bulk water is not available or continuous washing is not possible. Increased temperatures due to climate change thus presents a further challenge for optimal washing practices [92,93].

## 8 Research needs

A review of research methods relevant to the One Health concept revealed no methods suitable for controlling *Listeria* spp. and *L. monocytogenes.* Existing techniques for zoonotic infections do not address listeriosis adequately and fail to analyse contamination in diverse farm products, including organic and traditional products.

**Fig. 2:**
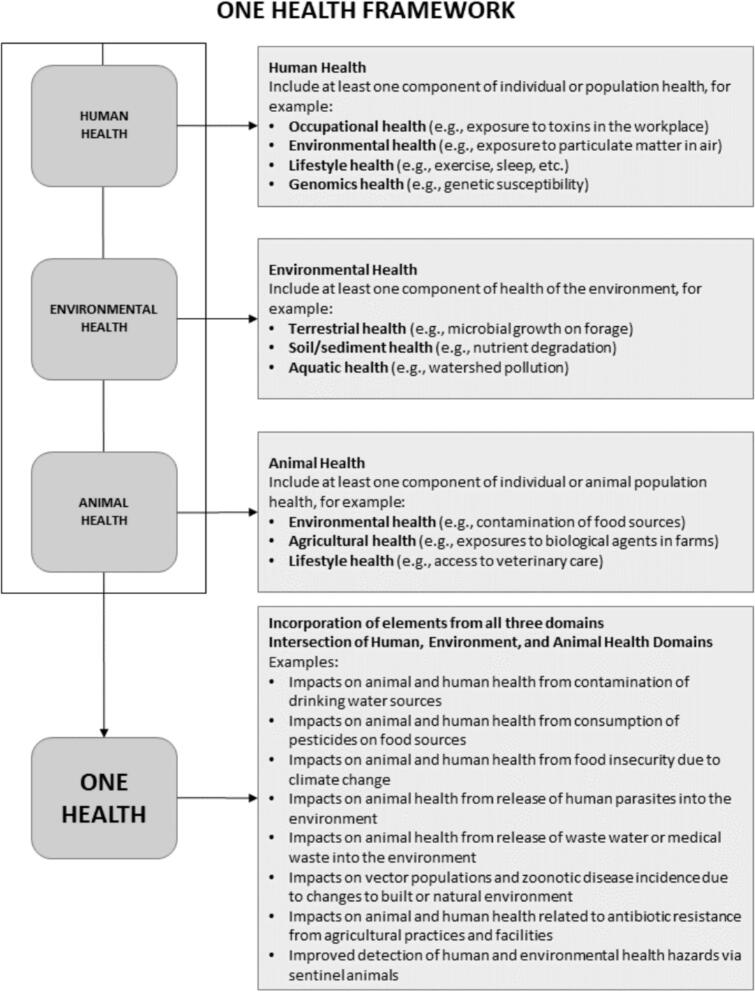
One Health Framework (modified according to Lebov et al., 2017 [94]).

This literature review led to the adaptation of methodologies previously described in other studies to align with our research questions on One Health Framework (Fig. 2.).

Addressing *Listeria* spp. contamination, and hence the safety of environmental products requires simultaneous examination of multiple domains within the One Health framework. *Listeria* spp., as a zoonotic pathogen, necessitates a comprehensive analysis of its habitats and potential contamination pathways. Understanding the risk factors associated with organic products involves microbiological and sociological research to examine the behaviours of high-risk groups. These groups include immunocompromised people, elderly individuals, pregnant women, and those requiring frequent hospitalization. Their exposure to listeriosis depends on regional endemicity, consumption patterns, water quality, seasonality, and fertilizer types.

Microbiological and sociological research methods should be combined to assess the risk of listeriosis infection in high-risk groups. Surveys targeting at-risk patients can be used to assess awareness of listeriosis, adherence to hygiene practices, and infection control measures. Microbiological methods can be used to test the hypothesis of chronic *Listeria* spp. carriage among people at risk. Microbiological methods can be used to study environmental contamination. First, environmental contamination in hospitals, particularly in cafeterias and food preparation areas, should be investigated.

Correlation studies should also be conducted to examine the relationships between *Listeria* spp. biofilms in hospitals and the knowledge levels of healthcare professionals. Behavioural characteristics, such as preferences for organic food, should be correlated with infection risk.

Infection pathways should also be investigated. Microbiological analysis of plants grown on farms and the same plants bought in markets and supermarkets should be conducted to determine the risks associated with plant and environmental contamination. Animal health is also a key issue, and microbiological analysis is needed to assess the impact of organic and conventional farming on animal products. Milk is a classical pathway for *L. monocytogenes* transmission and should be the focus of future studies.

## 9 Conclusion

This review highlights the pervasive and underestimated threat posed by *L. monocytogenes* across the interconnected domains of human, animal, plant, and environmental health. The One Health framework serves as an indispensable perspective for understanding and addressing the complex epidemiology and persistence of this pathogen, which continues to cause substantial morbidity and mortality worldwide. With its exceptional adaptability to environmental stressors, including extreme temperatures, high salinity, and desiccation, *L. monocytogenes* has established itself as a formidable zoonotic agent capable of surviving and proliferating across organic farming systems, food processing facilities, and diverse ecological niches influenced by human activity.

Human Health Impacts: Listeriosis, while relatively rare in the general population, remains among the deadliest foodborne infections, with case fatality rates exceeding 20–30 % in certain vulnerable groups. At-risk populations include pregnant women, neonates, the elderly, and individuals with compromised immune systems, whether due to chronic illnesses, cancer therapies, organ transplantation, or emerging widespread use of immunosuppressive biologics. The increasing life expectancy in many countries, coupled with higher rates of comorbidities requiring immunosuppressive treatments, is expanding the global pool of susceptible individuals. Cohort studies by Mylonakis et al., Charlier et al., and Coipan et al. underscore how listeriosis can manifest with severe invasive disease, including septicaemia and central nervous system infections, leading to lasting disabilities or death. Moreover, hospital outbreaks linked to contaminated ready-to-eat foods—such as sandwiches or salads—highlight ongoing gaps in infection control and food safety practices in healthcare settings, where vulnerable patients are concentrated.

Food Consumption Trends and Consumer Perception: There is a growing consumer preference for minimally processed, organic, or natural foods, which are often perceived as healthier. However, studies indicate that these foods may bypass certain decontamination steps, raising the risk of contamination with pathogens like *L. monocytogenes*.

Surveys show that many consumers are unaware of the risks associated with raw milk, unpasteurized cheeses, or improperly washed fresh produce, especially in immunocompromised individuals or during pregnancy, when *L. monocytogenes* can cause stillbirth, or neonatal infection. Targeted risk communication campaigns are essential to bridge this knowledge gap and promote safer food practices.

Farming Practices and Food Safety: Organic farming systems, despite clear ecological and biodiversity benefits, pose unique challenges in controlling *L. monocytogenes*. Practices such as the use of untreated manure, limited antimicrobial interventions, and exposure to wildlife can increase the likelihood of introducing and maintaining the pathogen in farm environments. Free-range systems, while improving animal welfare, may facilitate contact with wild birds and rodents, further expanding transmission routes. Conversely, conventional farming can contribute to pathogen persistence through overcrowded housing, biosecurity lapses, and routine antibiotic use, which risks selecting antimicrobial-resistant strains. Studies comparing organic and conventional farms have yielded inconsistent results, with some showing similar prevalence of *L. monocytogenes* and others indicating higher contamination in systems using organic fertilizers or composts. This variability emphasizes the importance of region-specific, large-scale research that considers farm size, animal species, climate, and manure management practices.

Environmental and Ecological Perspectives: The widespread environmental reservoirs of *L. monocytogenes*, including soil, surface water, irrigation systems, and biofilms in processing environments, make eradication challenging. The pathogen can survive for extended periods in manure-amended soils and persist on plant surfaces, especially when biofilms form on leaves or in processing equipment. Biofilm formation not only enhances environmental survival but also increases tolerance to disinfectants, contributing to repeated contamination cycles in food processing facilities. The role of complex soil microbiomes in suppressing or supporting *Listeria* spp. survival remains underexplored, representing a promising research area for developing natural, microbiome-based interventions. In addition, wild animals and synanthropic species like rodents and birds can spread *L. monocytogenes* between farms and processing environments, underlining the importance of wildlife control as part of farm biosecurity measures. Recent advances in nanotechnology offer promising tools for controlling *L. monocytogenes* in agricultural and food processing environments. Nanomaterials, such as silver, zinc oxide, and titanium dioxide nanoparticles, show strong antimicrobial and antibiofilm activity, reducing pathogen persistence on surfaces, packaging, and irrigation systems [95].

Climate Change and Emerging Risks: Climate change adds another layer of complexity to listeriosis control. Shifts in temperature, humidity, and precipitation patterns can influence the survival, growth, and spread of *L. monocytogenes*. Warmer temperatures can increase pathogen replication in soil and water, while extreme weather events like floods may mobilize pathogens from environmental reservoirs to agricultural fields. Droughts and water scarcity can compromise hygiene standards in food processing or preparation, particularly in low-resource settings. These dynamics highlight the need to integrate climate resilience into food safety planning, ensuring that preventive measures remain effective under changing environmental conditions.

Integration of One Health Pillars: Addressing *L. monocytogenes* effectively requires simultaneous consideration of human, animal, plant, and environmental health. Humans are exposed through the consumption of contaminated animal and plant products; animals can be asymptomatic carriers shedding the pathogen in feces or milk; plants can harbor *L. monocytogenes* on leaves and fruits after contamination from soil, manure, or irrigation; and environmental reservoirs like soil and water perpetuate the cycle. These interconnected pathways demonstrate that interventions limited to a single domain—such as food processing improvements—are insufficient without coordinated action across the entire food chain. Surveillance, risk assessments, and mitigation strategies must therefore be designed to integrate data and interventions across all One Health components.

## 10 Policy and research directions

• Strengthen Integrated Surveillance: Establish coordinated monitoring systems that simultaneously track *L. monocytogenes* prevalence in human cases, food products, farm environments, and wildlife, facilitating early detection and outbreak response.
• Advance Cross-Disciplinary Research: Promote studies combining microbiology, epidemiology, sociology, climate science, and ecology to identify context-specific risk factors and effective interventions.
• Enhance Hygiene and Processing Standards: Develop cleaning protocols targeting biofilms in food processing environments, with guidelines adaptable to both industrial and small-scale producers.
• Expand Public Education Initiatives: Launch culturally appropriate awareness campaigns highlighting the risks of consuming unpasteurized dairy, raw meats, and improperly washed produce, especially targeting pregnant women and the immunocompromised.
• Support Sustainable Farming Innovations: Encourage composting practices that reliably inactivate pathogens before manure application and explore the use of beneficial soil microbiota to suppress *L. monocytogenes* survival.
• Incorporate Climate Resilience: Adapt food safety guidelines to address risks arising from climate-related events, ensuring water quality and hygiene standards are maintained even under challenging environmental conditions.
• Foster International Collaboration: Promote harmonization of food safety standards and data sharing across borders to improve global responses to listeriosis outbreaks, which frequently involve multinational food supply chains.

The findings of this review demonstrate that *L. monocytogenes* is not only a local food safety issue but also a global public health challenge that requires integrated solutions. By embracing One Health principles—recognizing the interdependence of humans, animals, plants, and the environment—and implementing comprehensive, proactive strategies, we can reduce the burden of listeriosis, enhance food security, and contribute to more resilient and sustainable agricultural systems worldwide.

## Availability of data and materials

Data are accessible to researchers upon request for data sharing to the corresponding author.

## CRediT authorship contribution statement

**E. Ryzhova:** Writing – original draft, Conceptualization. **Wichmann Janine:** Writing – review & editing, Methodology, Formal analysis. **Howlett-Downing Chantelle:** Writing – review & editing, Formal analysis. **O. Holý:** Writing – review & editing, Writing – original draft, Supervision, Methodology, Formal analysis.

## Consent for publication

Not Applicable.

## Ethical approval and consent to participate

Not applicable.

## Funding

The work was supported by the project ‘Interdisciplinary Approaches to the Prevention and Diagnosis of Viral Diseases’ (CZ.02.01.01/00/23_021/0008856), funded by the 10.13039/501100008530European Regional Development Fund (ERDF) under the Johannes Amos Comenius Programme. We also acknowledge support from 10.13039/501100008530ERDF/ESF Project TECHSCALE (Grant CZ.02.01.01/00/22_008/0004587).

## Declaration of competing interest

The authors declare that they have no known competing financial interests or personal relationships that could have appeared to influence the work reported in this paper.

## Data Availability

Data will be made available on request.
